# The Treatment of Uterine Fibroids

**Published:** 1903-11-14

**Authors:** 


### THE TREATMENT OF UTERINE FIBROIDS.

Medical men have been paying a good deal of attention lately to the vexed question of the comparative merits of medical and surgical treatment in cases of fibroid tumour of the uterus. While it is doubtful if arbitrary rules of procedure can ever have any great value in this disease, seeing that there are so many important factors to be taken into consideration in each individual instance, the discussions which have taken place have produced one valuable result. The drawbacks to the various methods of treatment which may be employed have been brought into prominence so that their comparative merits and demerits can be to some extent estimated. This feature is well brought out in a paper by Mr. Alban Doran,[1] in which he makes a summary of our present knowledge with regard to the treatment of uterine fibroids. He does not attempt to lay down any hard and fast rules of practice for general use, but confines himself more or less to particulars.

He points out that a fibroid which is discovered accidentally and which is giving rise to no symptoms, does not need any immediate treatment, and may never require operative interference at all. But the future course of these tumours can never be foretold, and therefore the patient should be impressed with the necessity of keeping herself under the observation of a medical man.

One advantage of early operation may justly be urged, and that is the mental relief which follows the removal of the tumour. The very fact that a tumour is present, apart from the knowledge of possible complications, is a constant cause of mental worry, the exact amount of which will depend upon the temperament of the individual patient. A successful operation, therefore, usually means the restoration of mental comfort; but it involves many more benefits. The tumour being removed, the patient is safe from numerous troublesome and dangerous complications to which the growth might have given rise if it had not been extirpated; and the operator can feel fairly sure that a year later, had the operation been postponed to that date, its risks would have been greater. This assurance will apply far more certainly to each succeeding year, even for some time after the menopause, for the change of life does not necessarily entail the cure of fibroids. On the other hand, the physician who opposes operation when the tumour is unassociated with any complication may argue with some force that the growth of a fibroid is not deadly, as is the case with cancer; nor is it regular and certain to entail complications, one of which must at length prove fatal, as is the case with an ovarian tumour. The impossibility of calculating the future growth of a fibroid is put too much in the background by the advocates of hysterectomy. The operation itself is, at the best, no light experience, for even under the most skilful nursing the first few days are distressing to the patient. Hernia of the cicatrix is ever a possible after-result. Intestinal obstruction is always a danger whatever method of operation be adopted. Another sequel of hysterectomy, sometimes occurring a year or more after convalescence, is the discharge of ligatures in very inconvenient directions. The gravest possibility, however, when hysterectomy has been performed, is the occurrence of psychical disturbance. Hence, close inquiry should be made about the patient's previous history; and even when that has been done it may turn out that her friends have not revealed the truth. In this connection it should be borne in mind that hysterectomy for fibroid is decidedly more dangerous than double ovariotomy when the patient's mental condition is unsatisfactory.

Such are the chief arguments for and against operation when a patient has a fibroid which is not threatening to give trouble. Treatment, where an operation does not seem urgent, is purely symptomatic. When bleeding is present ergot is of use if given in moderate doses, unless cardiac disease is present, in which case ergot is contra-indicated. Chloride of calcium, though it has its advocates, is of doubtful utility. An abdominal belt, to support the tumour anteriorly, is always advisable. Certain authorities claim triumphs with electricity, but its application requires great experience, whilst the results are often doubtful and sometimes distinctly bad.

If operation be determined upon either because some complication is present or for some other reason, there are several different procedures to be considered, namely, oöphorectomy, myomectomy, vaginal hysterectomy, supra-vaginal hysterectomy, and panhysterectomy. With regard to the first it is an uncertain method. Mr. Doran has shown that, owing to the anatomical relations and the structure of the ovarian ligament, it is in most subjects impossible to avoid leaving some ovarian tissue behind. In some instances removal of one ovary only has been followed by cessation of the bleeding and shrinking of the tumour, while in other cases amputation of both appendages has been followed by no good result. He concludes, therefore, that the benefits obtained by oöphorectomy for fibroids are largely due to some other factor than removal of the ovaries, which factor has not yet been revealed. Myomectomy, when possible, is logically the best operation as it is the most conservative, but a good deal will depend upon the skill of the individual operator, as myomectomy is not satisfactory in the hands of the less experienced surgeon.

[1] Practitioner, November, 1903.

